# Ophthalmic Features and Implications of Poxviruses: Lessons from Clinical and Basic Research

**DOI:** 10.3390/microorganisms10122487

**Published:** 2022-12-15

**Authors:** Tolulope Fashina, Ye Huang, Joanne Thomas, Christopher D. Conrady, Steven Yeh

**Affiliations:** 1 Department of Ophthalmology and Visual Sciences, Stanley M. Truhlsen Eye Institute, University of Nebraska Medical Center, Omaha, NE 68105, USA; 2 Medical College of Georgia, Augusta University Medical Center, Augusta, GA 30912, USA; 3 Department of Pathology and Microbiology, University of Nebraska Medical Center, Omaha, NE 68105, USA

**Keywords:** monkeypox, poxvirus, orthopoxvirus, smallpox, vaccinia, ectromelia, camelpox, raccoonpox, skunkpox, volepox, emerging infectious diseases

## Abstract

Amidst the ongoing monkeypox outbreak, global awareness has been directed towards the prevention of viral transmission and case management, with the World Health Organization declaring the outbreak a public health emergency of international concern. Monkeypox virus is one of several species in the Orthopoxvirus genus, with other species of the genus including the variola, cowpox, mousepox, camelpox, raccoonpox, skunkpox, and volepox viruses. Although the nomenclature of these species is based on the animal host from which they were originally isolated, transmission from animals to humans has been reported with several species. The progression of disease, following an incubation period, typically consists of a prodromal phase with systemic flu-like symptoms. Various organ systems may be affected in addition to the formation of pathognomonic skin lesions. As monkeypox poses a continued public health concern, the ophthalmic sequelae of monkeypox virus, especially those leading to vision loss, warrant consideration as well. This review provides a comprehensive summary of the ophthalmic implications of poxviruses in clinical and laboratory settings reported in the literature, as well as areas of unmet need and future research.

## 1. Introduction (Orthopoxviruses)

Orthopoxvirus (OPV) is a genus of complex, double-stranded DNA viruses of the *Poxviridae* family. Several species are included in this genus, such as monkeypox virus and variola virus, the causative organism of smallpox. These species share similar characteristics, and many of these viruses have been shown to cause infection in humans, as seen in Table 1. Notably, these species are named based on which host they were first isolated, which does not necessarily indicate that this is the natural host for the virus [1]. Many in this group of viral pathogens can have a broad host range due to several biological traits, including a lack of specific receptors needed for entry, environmental stability of virions, and the virus harboring necessary genetic material for replication [2]. While cases of smallpox have decreased since vaccination efforts, monkeypox has made headlines as the World Health Organization (WHO) declared on 23 July 2022, that the multi-country outbreak of monkeypox was a global public health emergency [3]. Additionally, there have been reports of coinfections within OPV, including 151 cases of coinfection of monkeypox and varicella seen in the Democratic Republic of Congo through an active surveillance program from 2006 to 2007 [4]. Because of the broad range of hosts, recent widespread outbreaks of OPV, and the bioterrorism potential of certain viruses such as variola virus, OPV has emerged as a genus of interest for public health [5].

There are many routes of transmission to the host, including parenteral, respiratory, and mucosal [6]. The pathogenesis of OPV begins with penetration of the virus into the cell, which is not clearly understood. Most OPV species encode hemagglutinin, which is important for cell binding [7]. The virus then fuses with the cell membrane and releases the virus core, which has many components needed for initial replication [8]. Transcription is executed by the viral DNA-dependent RNA polymerase in the viral core. This replication occurs in the cytoplasm of the cell [6].

The diagnosis of OPV can be made through several methods. Electron microscopy of vesicle fluid or crusts is important in differentiating OPV from others, such as herpesvirus [6]. However, different species within OPV cannot be differentiated due to the lack of morphological differences [8]. There is also potential for cell culture cultivation with identification using antibodies or staining. Vero cells are a continuous cell line that can be used to propagate OPV and to monitor for cytopathic changes that could aid in detection [9]. Serology can be utilized to broadly identify OPV, but like electron microscopy, it cannot be used to differentiate species within the genus. The plaque reduction neutralization test is a specific serology method that has, however, been used to determine immune status by measuring neutralizing antibodies to specific species [10]. The primary way to identify species within OPV is through conventional polymerase chain reaction (PCR) or by assessing phylogeny through sequencing, but these methods can be time-consuming and require equipment that may not readily be available in all areas of outbreak. Real-time PCR can also be used to amplify part of the hemagglutinin gene to genotype the different OPV species [5]. The utility of multiplex real-time PCR has been shown in rapid detection and differentiation of various OPV strains [11]. While the similarities in the different species can complicate the diagnosis, many orthopoxviruses are cross-reactive and cross-protective, which can be beneficial.

The aim of this review is to provide a comprehensive summary of the clinical manifestations, route of transmission, vaccines, treatments, and ocular implications of eight OPV species: monkeypox, variola, cowpox, mousepox, camelpox, raccoonpox, skunkpox, and volepox virus. We also discuss postulated mechanisms for recent zoonotic outbreaks and appropriate protective measures to prevent transmission.

## 2. Monkeypox

Monkeypox virus (MPXV) is a zoonotic OPV that was first identified and isolated in 1958 following the shipment of infected monkeys from Singapore to Denmark [58]. It was, however, first diagnosed in a human suspected of having smallpox in 1970 [46].

### 2.1. Clinical Manifestations and Transmission

MPXV can be transmitted through animal-to-human transmission through direct contact with blood, body fluid, and mucocutaneous lesions of an infected animal [47]. Human-to-human transmission can also occur through close contact with body fluids, respiratory droplets, and skin lesions of an infected person [48]. Potential sexual exposure through genital and anal lesions has also been extensively reported in the literature [49,50,51,52,53,54,55,56,57,58,59,60]. The incubation period for MPXV varies from 5 to 21 days. The virus presents with generalized or localized mucocutaneous lesions that occur 1–3 days after sudden onset fever. Patients also present with associated headache, lymphadenopathy, myalgia, back pain, and asthenia [12,13].

### 2.2. Vaccine and Treatment

Several studies have reported that vaccination against smallpox is about 85% effective in the induction of cross-protection against MPXV. A two-dose vaccine based on a modified attenuated vaccinia virus (Ankara vaccine) was authorized to prevent monkeypox [13,18,19]. Two vaccines, ACAM200 and JYNNEOS™ (also known as Imvamune or Imvanex), are approved for use in the United States to prevent infection with orthopoxviruses smallpox and monkeypox. The Centers for Disease Control and Prevention (CDC) recommend vaccination for specific healthcare individuals at risk for exposure to the virus. They include clinical laboratory personnel who perform testing to diagnose orthopoxviruses, research laboratory personnel who directly handle infected specimens, and designated healthcare and public health response team members [23,61].

There are currently no antiviral drugs explicitly approved for the treatment of MPXV infection. The antiviral cidofovir has rarely been used as an off-label treatment for MPXV in humans and, although reported to be efficacious, further clinical investigation is necessary [20,21,22]. Tecovirimat, another antiviral that was designed and previously approved for the treatment of smallpox infection, was recently approved by the European Medical Association for the treatment of MPXV infection and is recommended in the treatment of severely ill individuals who are immunocompromised [23,24]. It has been an effective treatment that is generally well tolerated with minimal adverse effects [62,63,64,65].

### 2.3. Ocular Findings and Complications

Reports of ocular findings associated with MPXV infection include (a) periorbital, (b) conjunctival, and (c) corneal involvement. Individuals may report ocular redness, irritation, photosensitivity, discharge, and vision changes [66]. The characteristic vesicular rash seen in monkeypox may involve periorbital and orbital skin, and preseptal cellulitis has been reported [66,67,68]. Conjunctivitis was found to occur more commonly among patients with other symptoms present and may be a variable that is predictive of illness course [69]. Corneal scarring is the most common ocular sequela among survivors of the disease. During an outbreak in the Democratic Republic of Congo in 2003, an 8-year-old female was reported to have developed severe, sustained viral conjunctivitis of the left eye alongside corneal opacities, opaque pupil, and erythematous sclera six weeks after the onset of illness [14,15].

In a study that investigated the potential reservoir competence of the Gambian pouched rat (GPR) for MPXV by utilizing a combination of in vivo and in vitro methods, whitish-yellow corneal opacities and ocular discharge were noted in the eye of the inoculated rats 14 days post-infection. High levels of viral shedding were also noted in the conjunctival/corneal swabs taken from one of the rats [16].

Another study characterized a black-tailed prairie dog model of infection, featuring post-inoculation with the West African and Congo Basin strains of MPXV isolated during monkeypox outbreaks in 2003. Monkeypox viral DNA was detected in ocular swab samples 12 days post-infection, with periocular lesions also noticed 9–12 days post-infection in some animals [17].

## 3. Smallpox

Smallpox, caused by the variola virus (VARV), is described as one of the most lethal epidemic-causing diseases that resulted in an estimated 400 million deaths in the 20th century [70]. Although its origin remains unknown, humans are considered the only known hosts or reservoirs of smallpox. It was first reported in China, India, Southwest Asia, and the Mediterranean region between the 4th and 10th centuries. It was then observed in Europe in the 13th century and spread across the Americas, Southern Africa, and Australia between the 15th and 18th centuries. The *Variola minor* virus was later identified in South Africa, the United States, and South America in the 19th century [25,70,71]. Compared to the case fatality proportion (CFP) of *Variola major*, which was 5% to 25%, *Variola minor* was a less lethal form of the virus, with a CFP of <1% [72].

### 3.1. Clinical Manifestations and Transmission

Person-to-person transmission of VARV has been proven to occur through aerosols during close contact with infected persons [70,73]. It is characterized by a progressive skin rash, predominantly on the face and extremities. Following an incubation period of 7–19 days, a prodromal phase of infection occurs with associated fever, malaise, and headaches that last for about 2 to 4 days. This is followed by a maculopapular rash that progresses to a pustular rash and scabs that fall off and become permanent scars about three weeks after the initial rash appears [25,26].

### 3.2. Vaccine and Treatments

The variola vaccine was discovered in the late 18th century and helped achieve the global eradication of smallpox [34]. The original vaccine used was cowpox virus, which was eventually replaced with the less virulent vaccinia virus [35]. The U.S. Food and Drug Administration (FDA) has approved brincidofovir for the treatment of smallpox [36]. Brincidofovir has proven efficacy in poxvirus animal models as well [25]. Cidofovir has demonstrated utility in animal-based laboratory tests, although its use in treating smallpox has not been FDA-approved [36]. The antiviral tecovirimat has been shown to be efficacious in protecting mice from smallpox virus challenges [37,38]. The FDA has also approved tecovirimat in treating human smallpox disease [74]. Tecovirimat and cidofovir are both available options in the strategic national stockpile for use during public health emergencies [36].

### 3.3. Ocular Findings and Complications

Ocular findings associated with smallpox infection have been extensively documented and may involve anterior and posterior manifestations. As early as 1908, ocular involvement of smallpox was observed with (a) periorbital, (b) conjunctival, (c) corneal, and (d) posterior segment findings. Periorbital findings include rash, edema, and secretions. Conjunctival pathology consists of pustules and phlyctenules. Corneal involvement that presents as corneal ulceration is the most reported severe finding, with associated perforation with iris prolapse and hypopyon reported in some cases [27,28,29]. Smallpox virus was isolated in conjunctival swabs of patients with associated conjunctivitis who were being treated for the disease in India [75]. Posterior segment findings associated with smallpox infection include retinitis, chorioretinitis, and optic neuritis [29].

Although rare, cases of ocular vaccinia, which refers to eye infections resulting from accidental autoinoculation of the virus from the immunization site to the eye, have been reported. Ocular vaccinia commonly involves the eyelid, with occasional conjunctival and corneal involvement, which may include stromal opacification and scarring [30,31,32,33]. Posterior segment complications associated with ocular vaccinia include central retinal artery occlusion, pigmentary retinopathy, and chorioretinitis [29].

## 4. Cowpox

Cowpox (CPXV) is a rare zoonotic infection that can cause a self-limited disease, with more severe cases in the immunocompromised [41,76]. While cowpox virus and monkeypox virus both have a rodent reservoir, vaccinia virus and smallpox virus are pathogens that exclusively affect humans [77]. In recent years, there have been more frequent reports of humans with cowpox infection, mainly localized in Europe [78].

### 4.1. Clinical Manifestations and Transmission

The true reservoirs of CPXV are now considered to be wild rodents rather than cows. Incidental hosts include cows, zoo animals, cats, and humans [79]. CPXV transmission to humans from rodents or cats has been reported [80]. Cowpox typically has an incubation period of 8–12 days [39]. In humans, the disease may manifest as a painful macular lesion that becomes a hard black eschar with edema and erythema with associated findings including fever and lymphadenopathy [40]. Healing of skin lesions is characterized by scar formation. In those immunocompromised or with atopic predisposition, infection may be severe or lethal with dissemination of hemorrhagic pustules with areas of central necrosis and systemic manifestations such as fever [41].

An atypical clinical presentation of cowpox infection was reported in France in 2016. A smallpox-vaccinated patient developed thoracic varicella zoster-like-lesions following injury from a metallic guardrail previously stored in the ground. This occurrence stirred considerations for an alternative route of CPXV infection and the immunological status of smallpox-vaccinated patients for disease transmission [81].

A case of fetal death following maternal CPXV infection was documented in France in 2017, highlighting the risk for complications associated with orthopoxvirus infection during pregnancy [82].

### 4.2. Vaccine and Treatments

Due to similarities between the two viruses, the smallpox vaccine provides cross-protective immunity against cowpox virus [83]. Cidofovir has been used as a successful treatment in infected humans [84]. The efficacy of cidofovir, brincidofovir, and tecovirimat has been demonstrated in CPXV mouse models [85,86].

### 4.3. Ocular Findings and Complications

Ocular findings of cowpox include (a) periorbital, (b) conjunctival, and (c) corneal involvement. Reported cases of CPXV in humans include conjunctival chemosis and hyperemia progressing to keratitis and corneal ulceration, with severe cases requiring interventions such as amniotic membrane transplantation and autologous limbal stem cell transplantation [42,43]. Another individual affected by CPXV developed necrosis of the upper eyelid, keratitis, corneal opacity, and corneal neovascularization that necessitated multiple surgeries for treatment [44]. If ocular forms of cowpox are left untreated, progression to keratitis and corneal melting with corneal erosion may occur [45].

## 5. Ectromelia

Ectromelia virus (ECTV), also known as mousepox, closely resembles variola virus and monkeypox virus, two other members of the Orthopoxvirus genus: [87]. These similarities have made ECTV useful in small animal models to better understand smallpox pathogenesis [52,88]. ECTV infection has only been observed in mouse colonies maintained for research purposes, although it is believed that wild mouse populations in Europe may be colonized [89].

### 5.1. Clinical Manifestations and Transmission

Natural infection of ECTV is hypothesized to occur via microabrasions in the skin. The virus is speculated to initiate infection once it has reached the lower layers of the epidermis and dermis [90]. The incubation period typically lasts approximately six days, with a faster timeline of disease progression compared to smallpox [50]. Two forms of clinical mousepox have been observed, with the first being rapidly fatal and the second being a chronic disease characterized by lymphadenopathy and ulcerating skin lesions [51]. In the rapidly progressive version, clinical manifestations of ECTV in mice can be observed as early as three days after infection, including lethargy, mild respiratory distress, ocular abnormalities, and lesions at the base of ears and tails [52]. After a week, severe signs such as dyspnea may be seen.

### 5.2. Vaccine and Treatments

Vaccines and treatments specifically targeted toward ECTV eradication have not yet been closely studied in the literature.

### 5.3. Ocular Findings and Complications

Ophthalmic symptoms of ECTV include conjunctivitis and blepharitis [52]. The earliest ophthalmic signs included mild ocular symptoms such as excessive lacrimation three days after infection. Interestingly, there was an absence of virus-specific cytotoxic T lymphocytes (CTLs) in the conjunctivae for mice with ocular disease, while other diseased organs, such as the liver and spleen, had significantly higher levels of CTLs [51]. Increased cell apoptosis and an influx of lymphoid cells were seen in the conjunctiva of mice infected with the Moscow strain of ECTV [91]. Although ECTV infection among humans has not been reported in the literature, the ocular implications have been studied. Examination of human cornea epithelium in vitro revealed fusion and formation of giant cells within 1–2 weeks of culture with ECTV [92].

## 6. Camelpox

Camelpox virus (CMLV) causes a smallpox-like illness in the camel [54]. Camelpox outbreaks have had a devastating economic impact on populations that rely on camels as a resource [93].

### 6.1. Clinical Manifestations and Transmission

Transmission of CMLV can occur directly via inhalation or through a skin abrasion [94]. The virus can also be shed through secretions, resulting in indirect transmission [95]. An arthropod vector has also been hypothesized to play a role in spread [96]. Although camelpox has been most frequently studied among camels, there have been reported cases of humans developing mild skin lesions associated with camelpox [97]. Immunocompromised individuals may be particularly susceptible to CMLV, causing a more severe disease such as those seen with cowpox or monkeypox [53].

The incubation period of CMLV is 9–13 days [53]. The early stage of disease is characterized by fever, lymphadenopathy, and skin lesions. These lesions may present as erythematous macules and papules that form crusts when ruptured. Affected animals may also develop symptoms such as excess salivation, anorexia, increased lacrimation, and gastrointestinal distress [54]. Camels with severe disease may develop proliferative poxviral lesions in the bronchi [98].

### 6.2. Vaccine and Treatments

Both attenuated and inactivated forms of vaccination against CMLV are available for camels. Antiviral medications, most commonly cidofovir, can be used to treat infected animals and have a high level of efficacy [54].

### 6.3. Ocular Findings and Complications

Skin lesions may develop on the eyelids. Retinal lesions have also been reported and may lead to blindness [55].

## 7. Raccoonpox

Raccoons are the natural host of raccoonpox virus (RACV). There is little known about the epidemiology of RACV. However, RACV, along with skunkpox virus and volepox virus, is believed to be endemic to North America and, therefore, can be characterized as a “new world” orthopoxvirus [80].

### 7.1. Clinical Manifestations and Transmission

RACV was initially isolated from the respiratory tract of healthy-appearing raccoons in a swamp area in Maryland [99]. In this same geographic area, a seroprevalence of 23% was reported in the population of wild raccoons [100]. RACV has also been isolated from a skin lesion on a cat’s paw [56]. The clinical signs of raccoonpox are not fully understood but may involve development of minor and transient skin lesions with few significant symptoms [56].

### 7.2. Vaccine and Treatments

RACV is an effective vector for vaccine development due to its unique ability to trigger an immune response through mucosal routes [101]. There are no specific vaccines or treatments developed to prevent or treat RACV infection.

### 7.3. Ocular Findings and Complications

There are no reported ocular complications associated with RACV infection.

## 8. Skunkpox

Skunkpox virus (SKPV) is rare and has been tentatively designated a member of the Orthopoxvirus genus [102]. SKPV has only been isolated once from a skunk in Washington in 1978 [103].

Little is known about the specific clinical manifestations of SKPV, as it has not been closely studied in the laboratory or the field.

## 9. Volepox

Like SKPV and RACV, volepox virus (VPXV) is categorized as one of the three New World or North American orthopoxviruses. It was first isolated from a scab of an otherwise healthy vole in California [104].

### 9.1. Clinical Manifestations and Transmission

The species of voles and mice in which VPXV is found have minimal contact with humans, so there is believed to be a low chance for transmission [105]. VPXV has been incidentally found upon metagenomic next-generation sequencing to detect viral infections in kidney transplant recipients; however, the clinical significance is unclear [106]. Initial symptoms reported among mice infected with VPXV include skin lesions followed by decreased activity level, conjunctivitis, and facial edema. Internal organ necrosis was also seen in deceased or euthanized animals [57].

### 9.2. Vaccine and Treatments

There are no known vaccines or treatments specifically targeted toward VPXV eradication in the literature.

### 9.3. Ocular Findings and Complications

Specific ocular findings include maculae and papulae occurring on the eyelids, as well as conjunctivitis [57].

## 10. Special Considerations: Personal Protective Equipment (PPE) Recommendations

### 10.1. At Home

Measures can be taken to reduce the likelihood of monkeypox transmission in indoor settings. Hand hygiene should be performed after touching materials that have had contact with the monkeypox rash [107]. Infected individuals should wear a well-fitted mask, as should household members when they are near those who are sick. Attempts should be made to cover up exposed rashes on the skin, and gloves should be worn when in contact with others [107]. If able to do so, the infected individual should try to change bandages and handle contaminated materials independently while wearing gloves, followed by immediate handwashing [107]. Subsequently, all contaminated materials should be properly disposed of.

### 10.2. For the Healthcare Provider

Recommended PPE to be used by healthcare personnel includes a gown, gloves, goggles or face shield for eye protection, and a particulate respirator equipped with N95 filters or higher that has been approved by the National Institute for Occupational Safety and Health [108].

## 11. Special Considerations: Potential Explanations for Recent Orthopoxvirus Outbreaks

A number of explanations for recent outbreaks have been proposed. These include individual immunologic phenomena in humans as well as behavioral considerations.

### 11.1. Immunology

Vaccination with the vaccinia virus has been shown to have cross-protective immunity against other orthopoxviruses such as VARV, MPXV, and CPXV [109]. The specific immunologic mechanism underlying this phenomenon is multifactorial and may involve neutralizing antibodies [83]. Discontinuation of smallpox vaccinations in 1978 resulted in waning smallpox immunity and increased human susceptibility to contracting orthopoxvirus-related disease via animal-to-human transmission. Lower population herd immunity may then increase the likelihood of human-to-human transmission [78,110]. Unimmunized individuals who are infected have a greater risk of morbidity and mortality from infection as well [111].

### 11.2. Behavioral Factors

In addition to proposed immunologic mechanisms for recent orthopoxvirus outbreaks, human behavior is believed to be responsible, in part, for recent orthopoxvirus infections in multiple ways. Although not fully elucidated, there is evidence indicating that physical contact and exposure to fluids of an infected animal may increase the risk of disease [112]. A rise in ownership of exotic animals and livestock has contributed to increased international movement of zoonoses [113]. Transportation and abandonment of infected animals can release orthopoxviruses into naïve environments, resulting in outbreaks [113].

In particular, monkeypox transmission is closely associated with high-risk sexual behaviors. In the wake of the recent outbreak, a high proportion of monkeypox cases was noted in the men-who-have-sex-with-men (MSM) population [114,115]. Other behaviors, including multiple sexual partners in a short period, group sex, and gay and bisexual MSM, were also commonly observed among those infected individuals [114,116].

## 12. Conclusions

Although there may be variation between strains, orthopoxviruses characteristically present with skin lesions with progression from papules to vesicles to scabs. Other associated symptoms may include fever, lymphadenopathy, malaise, and arthralgia. However, less is known about the ocular manifestations of these viruses. Findings may consist of eyelid lesions, conjunctival or corneal involvement, and posterior segment findings such as retinitis, with particularly severe cases leading to devastating consequences such as permanent vision loss.

Outbreaks of zoonotic orthopoxvirus have occurred globally, the most notable being the ongoing monkeypox outbreak. Potential explanations for recent outbreaks include the population’s waning smallpox immunity and increased transportation of infected animals or fomites [117]. Future areas of focus should include transmission prevention measures, surveillance of spread, and recognition of symptoms. Identification of ophthalmic signs caused by poxviruses may assist the clinician in achieving a prompt diagnosis, which will be beneficial in protecting patients from vision loss when ophthalmic findings develop.

## Figures and Tables

**Table 1:** Summary of orthopoxvirus diseases with systemic and ophthalmic findings of clinical significance.

Pox Virus	Species Affected	Systemic Findings	Ophthalmic Findings and Vision Health Implications	Vaccine and Treatment
Monkeypox virus (MPXV)	Human Ape or monkey Gambian pouched rat (GPR) Prairie dog	Incubation period: 5–21 days Sudden-onset fever with mucocutaneous lesions 1–3 days later Headache, lymphadenopathy, myalgia, back pain, asthenia [12,13]	Human: corneal opacities, opaque pupil, erythematous sclera [14,15] GPR: corneal opacities and ocular discharge [16] Prairie dog: periocular lesions [17]	Vaccines: modified vaccinia vaccine, ACAM200, JYNNEOS^TM^ [13,18,19] Treatment: cidofovir (off-label) [20,21,22], tecovirimat to treat severely ill immunocompromised individuals [23,24]
Variola virus (VARV) or Smallpox	Human	Incubation period: 7–19 days Prodromal phase with fever, malaise, headaches for 2–4 days Maculopapular rash progressing to pustules and scabs that form scars [25,26]	Corneal ulceration with associated perforation, hypopyon, and iris prolapse [27,28,29] Posterior segment findings: retinitis, chorioretinitis, optic neuritis [29] Ocular vaccinia: conjunctival and corneal involvement or posterior segment complications [30,31,32,33]	Vaccine: made from live vaccinia [34] Treatment: cidofovir, brincidofovir, tecovirimat [35,36,37,38]
Cowpox virus (CPXV)	Human Cat Cattle Elephant Rodent	Incubation period: 8–12 days [39] Fever and lymphadenopathy Painful macular lesion that becomes hard black eschar with edema and erythema [40,41]	Eyelid necrosis Conjunctival chemosis and hyperemia Keratitis, corneal necrosis, corneal opacity, and corneal neovascularization with potential for corneal melt and erosion [42,43,44,45]	Vaccine: smallpox vaccine provides cross-protective immunity [46] Treatment: cidofovir [47], brincidofovir, tecovirimat [48,49]
Ectromelia virus (ECTV) or Mousepox	Human Mouse	Incubation period: approximately 6 days [50] May be rapidly fatal with symptoms including lethargy, respiratory distress, and skin lesions, or chronic disease with lymphadenopathy and ulcerating skin lesions [51,52]	Conjunctivitis, blepharitis, lacrimation [52]	NR
Camelpox virus (CMLV)	Human Camel	Incubation period: 9–13 days [53] Fever, lymphadenopathy, skin lesions [54]	Eyelid lesions and retinal lesions leading to blindness [55]	Vaccine: attenuated and inactivated forms
				Treatment: cidofovir [54]
Raccoonpox virus (RACV)	Raccoon Cat	Skin lesions with few significant symptoms [56]	NR	NR
Skunkpox virus (SKPV)	Skunk	NR	NR	NR
Volepox virus (VPXV)	Vole	Skin lesions, decreased activity level, facial edema, internal organ necrosis [57]	Maculae and papulae on eyelids; conjunctivitis [57]	NR

NR = not reported.

## Data Availability

Not applicable.
